# A new model to understand the complexity of inequalities in dementia

**DOI:** 10.1186/s12939-024-02245-w

**Published:** 2024-08-14

**Authors:** Clarissa Giebel

**Affiliations:** 1 Department of Primary Care & Mental Health, University of Liverpool, Liverpool, UK; 2 NIHR Applied Research Collaboration North West Coast, Liverpool, UK

**Keywords:** Dementia; inequalities, Barriers, Social care

## Abstract

Many people living with dementia and unpaid carers experience inequalities in care related to challenges in receiving a correct diagnosis, care and support. Whilst complexities of the evidence are well recognised including barriers in receiving a diagnosis or post-diagnostic care, no coherent model has captured the far-reaching types and levels of inequalities to date. Building on the established Dahlgren & Whitehead Rainbow model of health determinants, this paper introduces the new Dementia Inequalities model. The Dementia Inequalities model, similar to the original general rainbow model, categorises determinants of health and well-being in dementia into three layers: (1) Individual; (2) Social and community networks; and (3) Society and infrastructure. Each layer comprises of general determinants, which have been identified in the original model but also may be different in dementia, such as age (specifically referring to young- versus late-onset dementia) and ethnicity, as well as new dementia-specific determinants, such as rare dementia subtype, having an unpaid carer, and knowledge about dementia in the health and social care workforce. Each layer and its individual determinants are discussed referring to existing research and evidence syntheses in the field, arguing for the need of this new model. A total of 48 people with lived, caring, and professional experiences of dementia have been consulted in the process of the development of this model. The Dementia Inequalities model provides a coherent, evidence-based overview of inequalities in dementia diagnosis and care and can be used in health and social care, as well as in commissioning of care services, to support people living with dementia and their unpaid carers better and try and create more equity in diagnosis and care.

## Introduction

Dementia affects over 55 million people worldwide [1], with numbers continuously rising. As the disease progresses, people require increasing amounts of care and support, either by their family members and friends or externally via paid care. However, accessing care is not straightforward for many with dementia or their unpaid carers [17]. Equally, accessing and receiving a diagnosis in the first place can be fraught with difficulties [33].

Health inequalities are defined as unjust barriers to receiving equitable access to care, which could be avoided by the right interventions [39]. These include where people live, how they grow up, their educational and socio-economic background, and many other factors that can influence one’s health outcomes. These factors that are leading to inequalities in dementia are very similar, although also broader than those identified for the general population. When considering equitable dementia outcomes, we need to look at both receiving a diagnosis, and receiving adequate and suitable post-diagnostic care to live well and independently for as long as possible. There has been an increasing amount of evidence pointing to a myriad of factors leading to unequal outcomes in terms of both diagnosis and care [8, 15, 16, 27].

Whilst lack of available and accessible health care is generally higher in lower- and middle-income countries (LMICs), high-income countries are equally subject to inequalities. The majority of people with dementia live in LMICs [1], where receiving a diagnosis and/or post-diagnostic care are subject to increased barriers in countries such as India, Colombia, and Uganda [5]. This is due to a lack of infrastructure and investment into easily accessible care services, costs associated with accessing health care, as well as stigma and a lack of knowledge in the general population and often health care professionals [22]. Thus, fewer people receive a diagnosis of dementia and unpaid carers are often left with no knowledge about how to support their relative, with an expectation of providing care within the family. These barriers amplify the difficulties people with dementia (or a suspected, undiagnosed dementia), as well as their families, can experience, highlighting a need for a comprehensive model and overview of the various factors leading to inequalities in health and well-being for this growing population.

This paper sets out an advanced rainbow model of inequalities specifically for dementia – the Dementia Inequalities Model. Each layer of the rainbow, and its individual components (inequalities) are discussed in light of existing evidence in the field. With inequalities existing across the spectrum of dementia diagnosis and care, often intersecting, yet a lack of comprehensive model conceptualising and summarising these inequalities and their intersections, there is a clear need for such a new model. This model has the potential to be utilised in clinical and social care practice and commissioning, to support health and social care practitioners and commissioners to understand better and overcome the myriad of inequalities which many people with dementia and their unpaid carers experience.

### Overview of the original [10] model

The rainbow model of inequalities [10] breaks down the determinants of health inequalities into three layers. Starting at the core with individual-level factors such as age and gender, the second surrounding layer focuses on social and community network factors, followed by a third layer of general socio-economic, cultural and environmental conditions. The last layer includes work environment, education, and housing. The model has been used steadily since its inception to understand and address health inequalities [11], with issues inequalities raised in dementia fitting neatly into the different layers of inequalities that the general public can experience. Compared to other models of inequalities, such as Brunner and Marmot’s [6] Social Determinants of Health model, the rainbow model of inequalities by Dahlgreen and Whitehead [10] showcases individual, interlinked, factors on individual, interlinked, levels and thus provides a more nuanced understanding and detail of the different types of factors contributing to different health outcomes. In comparison, whilst Brunner and Marmot’s [6] model equally addresses how different factors influence diverse health outcomes, they are higher level than the two-tier approach evidenced in the rainbow model. For example, Brunner and Marmot [6] refer to material factors, social structure, health behaviour, and biological processes and their interlinkage. In contrast, Dahlgreen and Whitehead also refer to the social (and the societal) level, but furthermore detail specific factors within these levels, including education, living and working conditions, health care services, and housing. Thus, the rainbow model of inequalities provides a suitable foundation for conceptualising the determinants of inequalities in dementia.

### Public and stakeholder involvement

A total of 48 people with dementia, unpaid carers, health and social care professionals and Third Sector representatives were consulted about this model. This includes a series of public consultations with 40 stakeholders as part of the co-development of the Dementia Inequalities Game [19] (October 2022- June 2023), and an additional subsequent consultation with eight carers and Third Sector representatives (March 2024). People with lived, voluntary, and professional dementia experiences were reimbursed for their time.

As part of the Dementia Inequalities Game development, different people with dementia, unpaid carers, health and social care professionals, and Third sector representatives attended two remote and two in-person co-production and development workshops at the University of Liverpool. As part of these workshops, attendees were presented with an overview of the evidence of dementia inequalities, and asked to share and discuss their own experiences of barriers, and facilitators, to dementia diagnosis and care. In workshop 3, attendees were asked to do the same, as well as to discuss those highlighted in workshops 1 and 2 by previous attendees. This approach generated rich discussions and details about different types and layers of inequalities in dementia diagnosis and care, which have fed into a draft concept of this dementia inequalities model.

At the additional consultation in March 2024, eight unpaid carers and Third sector representatives were consulted remotely about the draft model. Feedback from all attendees at the meeting led to immediate modifications and additions of inequalities to the model during the meeting. At the end of the meeting, all attendees were asked whether the updated and modified model was representative of their experiences and their knowledge about other people’s experiences, with all in agreement. This additional layer of public consultation ensured that the model not only had all components previously identified, but also was visually and conceptually representing the inequalities experienced by people living with dementia and their unpaid carers.

### The Dementia Inequalities Model

**Fig. 1:**
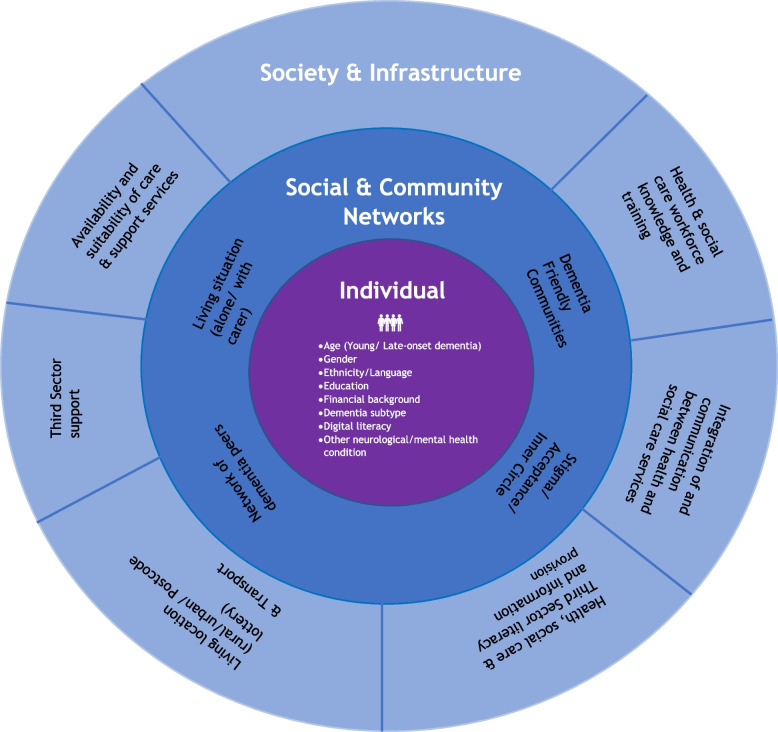
The Dementia Inequalities Model

The Dementia Inequalities Model builds on the existing Dahlgren & Whitehead [10] model of inequalities, and its three layers of inequalities – (i) Individual; (ii) Social & Community networks; and (iii) Society & Infrastructure. Each layer of the rainbow includes specific inequalities, which are evidenced below (see Fig. 1 for the model). Structuring individual inequalities across these three layers helps to provide a clearer framework and overview of the levels on which people with dementia and their unpaid carers can experience difficulties. Whilst inequalities are split into three layers, they also intersect within and across layers. People living with dementia and their unpaid carers rarely experience one issue alone that prevents them to access a timely and correct diagnosis or post-diagnostic care. Below, some examples of this intersectionality are highlighted and discussed. The issue of intersectionality is important to consider as trying to find solutions to improve access to diagnosis and care by addressing one inequality may result in little to no impacts.

#### Individual level

*Age* is a substantial barrier to both dementia diagnosis and care. People with (suspected) young-onset dementia (YOD), and thus below the age of 65 in high-income and 60 in lower- and middle-income countries, often experience delays in receiving a diagnosis and struggle findings suitable care services that are age-appropriate [8, 12, 17]. The delay in diagnosis and lack of subsequent care is also linked to a *lack of healthcare professional knowledge* [13, 29].

*Gender and educational background* have also been found to be linked to inequalities. Men with dementia for example were found to struggle to pay for social care during the cost of living crisis [18], whilst lower educational background is frequently linked to poorer outcomes in dementia [9].

People with dementia and carers from *minority ethnic backgrounds* or whose first language is not the country’s primary language can face difficulties in receiving a diagnosis and care [23, 27]. A growing evidence base highlights higher levels of *stigma* (see Social & Community Network level) and reluctance in non-White ethnic communities to approach a health care professional about symptoms, leading to delays or lack of a diagnosis [27]. Furthermore, care settings may not be culturally appropriate, requiring families to continue to take care of their relative with dementia [23]. This can also be reflected in cultural approaches to care, where external care is not sought out due to family caring duties and expectations [23], leaving some people with dementia and their unpaid carers at a lack of adequate support.

Having or caring for someone with a rarer* dementia subtype,* such as Lewy Body dementia, posterior cortical atrophy, or behavioural-variant fronto-temporal dementia, can cause an additional layer of difficulties in receiving a diagnosis and accessing suitable care. People with a rarer subtype have high levels of prior misdiagnosis and delayed diagnosis compared to those with Alzheimer’s Disease [3, 4, 33] and feel often inadequately supported with available services (see also Society & Infrastructure level).

With a particular increase in digitalisation since the COVID-19 pandemic, digital inequalities in dementia have been on the rise. Tujit et al. [36] noted particular barriers to primary care consultations for people with dementia, with changes to social care delivery in dementia during and since the pandemic evidenced repeatedly [7]. However, using digital technologies can also help connecting with others with dementia, albeit not everyone has the skills or technology to access care services or peer support remotely [34].

*Financial background*, including income, pension, and savings, can be a key barrier to accessing care. In England for example, in order to access subsidised care, people with dementia need to be aware of needing a Social Care Needs Assessment, provided by their Local Authority, which many are not aware of [20]. Considering that living in a care home is the most cost-intensive (paid) aspect of dementia care, many people may face delays in accessing full-time residential care although required.

An additional barrier to accessing diagnosis and care in dementia can be linked to *co-morbidities and sensory impairments.* Emerging evidence shows that for example people with a learning disability or those with hearing loss may face further challenges, as symptoms may be difficult to detect or instead attributed to the original diagnosis/impairment [25].

#### Social & Community networks level

*Living situation and carer availability*. Having an unpaid carer either living in (i.e. spouse/adult child) or close by to provide care with everyday activities and help to navigate the care system, appointments, and services can be a *substantial* benefit. People with dementia can often be at a disadvantage without having an unpaid carer, as carers can help to find external care and support and help with legal aspects surrounding the diagnosis also. This is particularly the case where adult child carers may be more digitally literate than their parent with dementia, representing a common inequality and often referred to as the digital gap [34].

*Stigma* can be a substantial barrier in both family and peer networks, as well as broader within the community, and can hinder people from seeking help for symptoms and from accessing suitable care [26]. This can be particularly pronounced in people from non-White ethnic backgrounds [23], and in lower- and middle-income countries such as Brazil, India, and Pakistan [2, 14, 35]. In Pakistan for example, family members are reluctant to seek help if they have sufficient knowledge about the symptoms of dementia in the first place [2]. An approach to reducing stigma are designated and promoted *Dementia Friendly Communities,* which involve increased knowledge in shops and other organisations to help people with dementia and their carers navigate their daily lives within their community easier [31].

Where health and social are infrastructure may fail, having a* network of dementia peers* can often help to cope with the experiences of dementia and caregiving, and can help signpost to information and services.

#### Society & Infrastructure level

Living location (*rural/urban/peri-urban/postcode lottery)* can affect access to diagnosis and care. People living in more rural regions can face difficulties in seeing a GP for their symptoms and receiving a diagnosis whilst also facing challenges in accessing suitable post-diagnostic care [16, 30]. This can be influenced by factors of transport, especially lack of adequate public transport, as well as intersecting with lack of suitable services for people with rarer subtypes of dementia, or young-onset dementia, and thus specific caring needs [8]. However, people with dementia and carers can also face challenges in urban areas [37]. Accessing a diagnosis and suitable care is often described as a postcode lottery, whereby living in one street or postcode may facilitate, or impede, access depending on coverage of NHS and social care services.

Living location also matters on a global scale. Whilst inequalities are evident in high-income countries, LMICs can represent particular systematic challenges for dementia diagnosis and care. This is influenced by cultural attitudes towards dementia and the associated stigma, lack of resources and health services, lack of social care, and cultural and societal expectations to provide care within the family as opposed to seeking out external care and support [2, 5]. There are many overlaps in inequalities between high and low-and middle-income countries, such as lack of workforce knowledge, availability of carer (social and community level), and lack of information provision from care providers. However, Third sector availability be me more limited in LMICs for example, or suitability of support services may not be relevant as little to no services for dementia exist.

Issues surrounding *availability and suitability of services*, including day care and home care, intersect with where people live and their dementia subtypes and age. Some regions, particularly more urban centres, provide greater availability of services, with availability also influenced by the local and regional investment by local councils, health and social care, and Third Sector [30]. Even where sufficient numbers of services are provided, services may not be suitable as they fail to provide adequate care to people with for example Lewy Body or semantic dementia, whilst people with young-onset dementia and their carers may not wish to join groups which provide activities targeted at older adults [8].

People with dementia and unpaid carers often report that *health and social care professionals lack adequate training, knowledge, and skills* about dementia, different subtypes, and service navigation. This also links in with the factor of age and rare dementia subtype [13, 32]. This is linked to a *lack of suitable information* provided about symptom, caring, legal, and support aspects of dementia provided after a diagnosis, in addition to a lack of integration between health and social care [16]. As a result, once someone receives a diagnosis from a health care professional, they are often lost in the system as there is often no clear referral pathway to social care. To overcome these shortcomings, *Third Sector support* is a vital point of call for people with dementia and unpaid carers to receive information and support [38].

## Discussion

This is an overview of the complex inequalities that people with dementia and their unpaid carers can face in the diagnostic and post-diagnostic care journey, illustrated in the new Dementia Inequalities Model. These inequalities are often interlinked, such as living with a rare dementia subtype (individual level), availability and suitability of care and support (society and infrastructure level) and lack of health and social care workforce knowledge and training (society and infrastructure level). Thus, finding solutions to address one barrier to diagnosis and care may intersect with other barriers also.

Whilst the number and range of inequalities in dementia diagnosis and care is vast, solutions are starting to emerge. These include Dementia Care Navigators and Admiral Nurses, who help people with dementia and their families after a diagnosis with navigating the complex care system and providing targeted support [21], as well as improving joint working and developing opportunities for service development [38]. To address these inequalities, a broader lens needs to be applied to understand the intersection of various types and levels of inequalities, including how people with dementia can be affected at an individual, social, and societal level. Whilst the original Dahlgreen & Whitehead [10] model outlined different factors leading to health inequalities for the general population, this new model adds dementia-specific elements which are not relevant to the general population. These include young-onset dementia, rare dementia subtype, having a caregiver, and stigma, and Third sector support, among others. People with dementia and their carers face some unique challenges compared to the general population, warranting the need for this specific model on inequalities. Having one conceptual framework that ties together these types and layers of inequalities is important considering that most research focuses on individual barriers, such as ethnicity or age, in relation to dementia diagnosis or care.

This model also brings together diverse experiences of inequalities across the globe and different country and cultural settings. Care provision in LMICs often differs greatly from that in high-income countries, whilst there also remain significant variations in care provision across and within high-income countries [19, 20]. The lack of care infrastructure, the need to pay for care, and lack of knowledge about dementia in the workforce and general population can place additional barriers to people with a suspected or a diagnosis of dementia, and their family carers, living in LMICs (i.e. [1, 28]). This is in addition to the stigma often experienced surrounding dementia and mental health issues [5], linked to the lack of knowledge about the condition. This model can help to clearly showcase to care professionals, local community leaders, and local and national governments the issues that people with dementia and their families are facing, as increasing knowledge about dementia is still an urgent priority for different LMICs [24].

## Conclusions

Having a dedicated model on dementia inequalities clearly stresses the importance of issues unique to the growing population and their carers. This model and overview can further help provide an understanding of the many shapes of inequalities and provide guidance for commissioners and health and social are professionals on how diagnosis and care can be made more equitable for people with dementia and their carers. The model also provides an urgent framework and overview of the intersection of inequalities in dementia diagnosis and care for future research, to allow the exploration of how inequalities are linked as opposed to focusing solely on one factor.

## Data Availability

No data were collected as part of this model development.

## Data availability

No datasets were generated or analysed during the current study.
